# Note on a Weighted Geometric Inequality in Hyperbolic Space

**DOI:** 10.1007/s12220-026-02565-z

**Published:** 2026-08-12

**Authors:** Botong Xu

**Affiliations:** Department of Mathematics, Technion-Israel Institute of Technology, 32000 Haifa, Israel

**Keywords:** Horospherically convex, Locally constrained curvature flow, Alexandrov–Fenchel inequalities, Hyperbolic space, 53C44, 52A55

## Abstract

In this paper, we prove that the solution to a locally constrained flow in hyperbolic space introduced by Hu et al. [14] exists for a long time and converges exponentially fast to a geodesic sphere centered at the origin if the initial hypersurface is strictly horo-convex. Unlike previous results, we do not require the initial hypersurface to be star-shaped. As a consequence, we refine an Alexandrov–Fenchel type weighted geometric inequality proved by them so that both sides are isometry-invariant.

## Introduction

Let $\mathbb {H}^{n+1}$ denote the (n+1)-dimensional hyperbolic space, and let *r* denote the distance function from a given point $\mathcal {O}$ in $\mathbb {H}^{n+1}$. Then $\mathbb {H}^{n+1}$ can be viewed as a warped product manifold $[0, \infty ) \times \mathbb {S}^n$ equipped with metric $\bar{g} = dr^2 + \sinh ^2 r g_{\mathbb {S}^n}$ so that $\mathcal {O} = \left\{ X \in \mathbb {H}^{n+1};~r \left( X\right) =0\right\}$. Here $\mathbb {S}^n$ denotes the unit sphere.

Let Ω be a bounded domain with smooth boundary $M = \partial \Omega$ in $\mathbb {H}^{n+1}$. We say Ω (or *M*) is horo-convex (h-convex for short) if the principal curvatures are at least 1 everywhere on *M*. We say *M* is strictly h-convex if the principal curvatures are greater than 1. Compared to general convex domains in hyperbolic space, h-convex domains share greater similarities with Euclidean convex bodies, leading to extensive research in recent decades (see, e.g., [2, 5, 14, 16, 17, 21, 22, 28]).

For horo-convex domains in $\mathbb {H}^{n+1}$, Andrews et al. [2] introduced a natural sequence of functionals called modified quermassintegrals $\{ \widetilde{W}_m\}_{m=0}^n$, see (2.8). They established that, for given integers $0 \le m < \ell \le n$, the following Alexandrov–Fenchel type inequality holds for any strictly h-convex bounded domain Ω:

1.1 $$\begin{aligned} \widetilde{W}_{\ell } \left( \Omega \right) \ge \tilde{f}_{\ell } \circ \tilde{f}_m^{-1} \left( \widetilde{W}_m \left( \Omega \right) \right) , \end{aligned}$$

with equality if and only if Ω is a geodesic ball. Here $\tilde{f}_m: \mathbb {R}_+ \rightarrow \mathbb {R}_+$ is defined by $\tilde{f}_m \left( r\right) = \widetilde{W}_m \left( B\left( r\right) \right)$, i.e., the *m*th modified quermassintegral of a geodesic ball of radius *r* in $\mathbb {H}^{n+1}$. Later, Hu et al. [14] gave an alternative proof of (1.1) by introducing and studying the following locally constrained curvature flow for a family of h-convex hypersurfaces $X: M \times [0,T) \rightarrow \mathbb {H}^{n+1}$.

1.2 $$\begin{aligned} \frac{\partial }{\partial t} X \left( x, t\right) = \left( \left( \cosh r-\tilde{u} \right) \frac{p_{m-1}(\tilde{\kappa })}{p_m \left( \tilde{\kappa }\right) } -\tilde{u}\right) \nu \left( x, t\right) , \quad m =1, \ldots , n. \quad \end{aligned}$$

Let us address the notation that appears in the flow (1.2). For any hypersurface *M* in $\mathbb {H}^{n+1}$, we denote by ν the unit outer normal of *M* and by $\kappa = \left( \kappa _1, \ldots , \kappa _n\right)$ the principal curvatures of *M*. We set $\tilde{u} (X):= \langle \sinh r \partial _r, \nu (X) \rangle$ for X∈M and denote $\tilde{\kappa }_i = \kappa _i-1$. Moreover, we denote by $p_k$, k=0,…,n, the *k*th normalized elementary symmetric polynomial in *n* variables. Hu et al. [14] showed that if the initial hypersurface $M_0 = \partial \Omega _0$ is strictly h-convex with $\mathcal {O} \in \Omega _0$, then the flow (1.2) exists for all time and $M_t$ converges to a sphere centered at the origin $\mathcal {O}$ exponentially fast as $t \rightarrow \infty$. They then gave a new proof of the inequality (1.1) by showing that $\widetilde{W}_m \left( \Omega _t\right)$ is preserved and that $\widetilde{W}_{m+1} \left( \Omega _t\right)$ is non-increasing along the flow (1.2). Using the flow (1.2), they also established the following Alexandrov–Fenchel type weighted geometric inequality in hyperbolic space.

Before stating their result, we recall that a smooth hypersurface *M* in $\mathbb {H}^{n+1}$ is called star-shaped if there exists a smooth positive function *r* on the unit sphere $\mathbb {S}^n$ such that

1.3 $$\begin{aligned} M = \left\{ \left( r \left( \theta \right) , \theta \right) \in \mathbb {R}_+ \times \mathbb {S}^n;~ \theta \in \mathbb {S}^n \right\} . \end{aligned}$$

Moreover, we note that if Ω is an h-convex domain containing the origin $\mathcal {O}$, then $M = \partial \Omega$ is star-shaped.

### Theorem A

([14]) Let $M = \partial \Omega$ be a smooth, star-shaped, strictly h-convex hypersurface in $\mathbb {H}^{n+1}$. Then for any m=1,…,n-1, there holds

1.4 $$\begin{aligned} \int _M \left( \cosh r -\tilde{u}\right) p_m \left( \tilde{\kappa }\right) \, d\mu \ge \tilde{h}_m \circ \tilde{f}_m^{-1} \left( \widetilde{W}_m \left( \Omega \right) \right) , \end{aligned}$$

where $\widetilde{W}_m \left( \Omega \right)$ is the *m*th modified quermassintegral of Ω, $\tilde{f}_m(s):= \widetilde{W}_m \left( B\left( s\right) \right) ,$ and $\tilde{h}_m (s):= \left| \mathbb {S}^n\right| \left( \cosh s-\sinh s\right) ^{m+1} \left( \sinh s\right) ^{n-m}$. Equality holds in (1.4) if and only if *M* is a geodesic sphere centered at the origin.

### Remark 1.1

1. Indeed, Hu et al. [14] proved Theorem A for smooth, star-shaped, h-convex hypersurfaces with the shifted principal curvatures $\tilde{\kappa }_i$ lying in the *m*th Garding cone $\Gamma _m^+$ defined by
  $$\begin{aligned} \Gamma _m^+ = \left\{ \alpha \in \mathbb {R}^n;~ p_i \left( \alpha \right) >0, \ i=1, \ldots , m \right\} . \end{aligned}$$
  This convex assumption on *M* is slightly weaker than strict h-convexity.
2. The star-shaped condition of the initial hypersurface is standard in the study of locally constrained curvature flows and was used by default in [14] to establish the a priori estimates of the flow (1.2).
3. The author with Li [18] showed that the inequality (1.4) holds for all bounded domains with $C^1$ boundary in $\mathbb {H}^{n+1}$ for the remaining case m=0.

It is not hard to see that, unlike the right-hand side of (1.4), the left-hand side depends on the relative position between Ω and the origin $\mathcal {O}$, and is therefore not invariant under the isometries of $\mathbb {H}^{n+1}$. Our motivation for this paper is to refine the inequality (1.4) so that both sides are independent of the position of the origin. The idea is to first remove the star-shaped assumption on *M* in (1.4). Next, we fix Ω and find a suitable new origin (possibly outside Ω) that minimizes the left-hand side of (1.4), which will induce an isometry-invariant geometric quantity of Ω. Finally, we can replace the left-hand side of (1.4) with this new quantity.

To this end, our first theorem shows that, as long as the initial hypersurface $M_0 = \partial \Omega _0$ is strictly h-convex, the existence and convergence results of the flow (1.2) established by Hu et al. [14, Theorem 1.6] still hold without assuming $\mathcal {O} \in \Omega _0$.

### Theorem 1.1

Let $X_0: M^n \rightarrow \mathbb {H}^{n+1}$ $\left( n \ge 2\right)$ be a smooth embedding such that $M_0 = X_0 \left( M\right)$ is a smooth, strictly h-convex hypersurface in $\mathbb {H}^{n+1}$. Then the flow (1.2) has a smooth solution for all time $t \in [0, \infty ),$ and $M_t$ is strictly h-convex for any t>0 and converges smoothly and exponentially to a geodesic sphere of radius $r_{\infty }$ centered at the origin as $t \rightarrow \infty ,$ where $r_{\infty }$ is determined by $\widetilde{W}_m \left( B \left( r_{\infty }\right) \right) = \widetilde{W}_m \left( \Omega _0\right)$.

We also mention the recent work by Pan and Scheuer [23], who studied a related curvature flow in the hemisphere without the star-shaped assumption on the initial hypersurface.

To formulate the inequality that will be obtained by applying Theorem 1.1, let us recall the hyperboloid model of $\mathbb {H}^{n+1}$. Denote by $\mathbb {R}^{n+1,1}$ the (n+2)-dimensional Minkowski space equipped with the Lorentzian product $\langle \cdot , \cdot \rangle _1$, such that for any $X = \left( x, x_{n+2}\right) \in \mathbb {R}^{n+1,1}$, it holds $\langle X, X \rangle _1= \left| x\right| ^2 - x_{n+2}^2$. For any timelike vector *X*, we define its hyperbolic length (also known as proper time) *N*(*X*) as

$$\begin{aligned} N \left( X\right) = \sqrt{-\langle X,X \rangle _1}. \end{aligned}$$

Apparently, $N\left( X\right) \le \left| x_{n+2}\right|$. Throughout this paper, we view $\mathbb {H}^{n+1}$ as a hyperboloid in $\mathbb {R}^{n+1,1}$:

$$\begin{aligned} \mathbb {H}^{n+1} := \left\{ X= \left( x, x_{n+2}\right) \in \mathbb {R}^{n+1,1};~\langle X, X \rangle _1 = -1, \ x_{n+2}>0\right\} . \end{aligned}$$

Using Theorem 1.1, we can establish the following geometric inequality.

### Theorem 1.2

Let $M = \partial \Omega$ be a smooth, strictly h-convex hypersurface in $\mathbb {H}^{n+1}$. Then for any m=1,…,n-1, there holds

1.5 $$\begin{aligned} N \left( \int _M \left( X- \nu \right) p_m \left( \tilde{\kappa }\right) \, d\mu \right) \ge \tilde{h}_m \circ \tilde{f}_m^{-1} \left( \widetilde{W}_m \left( \Omega \right) \right) . \end{aligned}$$

Equality holds in (1.5) if and only if *M* is a geodesic sphere.

### Remark 1.2

Both sides of the inequality (1.5) do not depend on the relative position between Ω and $\mathcal {O}$.

Now we explain in what sense Theorem 1.2 provides a refinement of Theorem A. By setting $E_{n+2} = (0, 1)$ as the origin $\mathcal {O}$ of $\mathbb {H}^{n+1}$, it can be verified that, at any X∈M, the last coordinate of X-ν is precisely $\cosh r - \tilde{u}$, see (2.5). Then the left-hand side of (1.4) can be expressed as

$$\begin{aligned} - \left\langle \int _M \left( X-\nu \right) p_m \left( \tilde{\kappa }\right) \, d\mu , E_{n+2} \right\rangle _1. \end{aligned}$$

Since X-ν lies on the future light cone for any $X \in \partial \Omega$ and $\tilde{\kappa } >0$ on *M*, as a conic combination of future lightlike vectors, we know that $\int _M \left( X-\nu \right) p_m \left( \tilde{\kappa }\right) \, d\mu$ is future timelike. This implies

$$\begin{aligned} \int _M \left( \cosh r -\tilde{u}\right) p_m \left( \tilde{\kappa }\right) \, d\mu \ge N \left( \int _M \left( X-\nu \right) p_m \left( \tilde{\kappa }\right) \, d\mu \right) . \end{aligned}$$

Consequently, Theorem 1.2 refines Theorem A.

Denote by $d_{\mathbb {H}^{n+1}} \left( \cdot , \cdot \right)$ the distance function between two points in $\mathbb {H}^{n+1}$. The following corollary of Theorem 1.2 shows how Theorem 1.2 improves Theorem A when the domain Ω is symmetric with respect to a point in $\mathbb {H}^{n+1}$.

### Corollary 1.1

Let Ω be a bounded domain with smooth, strictly h-convex boundary $M= \partial \Omega$ in $\mathbb {H}^{n+1}$. Assume that Ω is symmetric with respect to a point *Y* in $\mathbb {H}^{n+1}$. Then

1.6 $$\begin{aligned} \frac{1}{\cosh d_{\mathbb {H}^{n+1}} \left( \mathcal {O}, Y\right) }\int _{M} \left( \cosh r- \tilde{u}\right) p_m \left( \tilde{\kappa }\right) \, d\mu \ge \tilde{h}_m \circ \tilde{f}_m^{-1} \left( \widetilde{W}_m \left( \Omega \right) \right) . \end{aligned}$$

Equality holds if and only if Ω is a geodesic ball centered at *Y*.

In the next part of this section, we discuss another type of weighted geometric inequality in $\mathbb {H}^{n+1}$. Let Ω be a bounded domain with convex boundary $M = \partial \Omega$ in $\mathbb {H}^{n+1}$. Note that there are weighted geometric inequalities in $\mathbb {H}^{n+1}$ that estimate the weighted curvature integral

$$\begin{aligned} \int _{M} \cosh r F \left( \kappa \right) \, d\mu \end{aligned}$$

from below by an isometry-invariant geometric quantity, where *F* is a positive function depending only on the principal curvatures; see e.g., [7, 8, 30]. In some cases, the hypersurface *M* is assumed to be star-shaped with respect to the origin $\mathcal {O}$, e.g., [7]. In the following, we provide a simple observation that may help remove such an assumption when *M* is convex.

### Proposition 1.1

Let Ω be a convex body with boundary $M= \partial \Omega$ in $\mathbb {H}^{n+1},$ and let $\mathscr {F}$ be a positive function on *M*. Then there exists a point $X_0 \in \Omega$ such that

1.7 $$\begin{aligned} \frac{1}{\cosh d_{\mathbb {H}^{n+1}} \left( \mathcal {O}, X_0\right) }\int _M \cosh r \mathscr {F} \, d\mu = \int _M \cosh d_{\mathbb {H}^{n+1}}\left( X, X_0\right) \mathscr {F} \, d\mu . \end{aligned}$$

For example, Hu et al. [14, Theorem 1.4] proved the following Alexandrov–Fenchel type inequality for h-convex domains in $\mathbb {H}^{n+1}$:

1.8 $$\begin{aligned} \int _M \cosh r p_k \left( \kappa \right) \, d\mu \ge h_k \circ f_m^{-1} \left( W_m \left( \Omega \right) \right) , \quad 1\le k \le n, \quad 0\le m\le k. \end{aligned}$$

Here $f_m: \mathbb {R}_+ \rightarrow \mathbb {R}_+$ is defined by $f_m \left( s\right) = W_m \left( B(s)\right)$, i.e., the *m*th quermassintegral of a geodesic ball of radius *s* in $\mathbb {H}^{n+1}$, see (2.7); and $h_k$ is a function that guarantees that equality holds in (1.8) whenever Ω is a geodesic ball centered at the origin $\mathcal {O}$.

As a standard assumption, it was implicitly assumed in the inequality that $\mathcal {O} \in \Omega$. However, Proposition 1.1 shows that this assumption can be removed.

In the last part of this section, we provide a historical remark related to the work in this paper. The Alexandrov–Fenchel inequality is fundamental in the Brunn–Minkowski theory for convex bodies in Euclidean space. Special cases of it compare different orders of quermassintegrals of convex bodies. Guan and Li [10] showed that these inequalities still hold under the assumption that the domain is star-shaped and *k*-convex, which is weaker than classical convexity, by introducing a locally constrained curvature flow. A remarkable feature of this technique is that it can be generalized to prove Alexandrov–Fenchel type inequalities in various non-Euclidean spaces (e.g., [6, 11–13, 19, 26, 29]), and it can be applied to establish Alexandrov–Fenchel type inequalities for hypersurfaces with boundaries (e.g., [15, 20, 27]).

In recent work with Li [17], the author attempted to develop a Brunn–Minkowski type theory for h-convex domains in $\mathbb {H}^{n+1}$ by introducing a new summation of domains in $\mathbb {H}^{n+1}$ called the hyperbolic *p*-sum, and found that both inequalities (1.1) and (1.4) can be viewed as special cases of a conjectured Minkowski type inequality induced by the summation. Meanwhile, it was implicitly conjectured in [17, Conjecture 10.6] that the star-shaped assumption of *M* in (1.4) is unnecessary. This serves as additional motivation for us to prove Theorem 1.2. However, to ensure consistency, a detailed discussion will be presented elsewhere.

The rest of this paper is organized as follows. In Sect. 2, we collect the properties of symmetric functions and study h-convex hypersurfaces in $\mathbb {H}^{n+1}$. In Sect. 3, we derive the evolution equations along a scalar equation determined by the flow (1.2). In Sect. 4, we provide the explicit solution to the flow (1.2) when the initial hypersurface is a geodesic sphere. In Sect. 5, we give the a priori estimates of the flow (1.2). In Sect. 6, we prove Theorems 1.1 and 1.2, Corollary 1.1 and Proposition 1.1.

## Preliminaries

### Symmetric Functions

In the following, we collect some notations and properties of symmetric functions; see e.g., [3, 9] for a detailed discussion.

Given a symmetric function $G: \mathbb {R}^n \rightarrow \mathbb {R}$, one can define an induced function $\textbf{G}$ on n×n symmetric matrices by setting $\textbf{G} \left( A\right) = G \left( \alpha \left( A\right) \right)$, where $\alpha \left( A\right) = \left( \alpha _1, \ldots , \alpha _n\right)$ are the eigenvalues of *A*. Denote

$$\begin{aligned} \dot{G}^i \left( \alpha \right) = \frac{\partial G}{\partial \alpha _i}, \quad \ddot{G}^{ij} \left( \alpha \right) = \frac{\partial ^2 G}{\partial \alpha _j \partial \alpha _i},\quad \dot{\textbf{G} }^{ij} \left( A\right) = \frac{\partial \textbf{G}}{\partial A_{ij}},\quad \ddot{\textbf{G}}^{ij,kl} \left( A\right) := \frac{\partial ^2 \textbf{G}}{\partial A_{kl} \partial A_{ij}}. \end{aligned}$$

Whenever $A = \text {diag} \left( \alpha _1, \ldots , \alpha _n\right)$ is a diagonal matrix, one has $\dot{G}^i \left( \alpha \right) = \dot{\textbf{G}}^{ii} \left( A\right)$ and $\ddot{G}^{ij} \left( \alpha \right) = \ddot{\textbf{G}}^{ii, jj} \left( A\right)$. In the sequel, we write $\textbf{G}$ as *G* if there is no confusion.

For any integer $k \in \left\{ 1,2,\ldots , n\right\}$, the *k*th normalized elementary symmetric polynomial $p_k$ is a symmetric function of *n* variables defined by

2.1 $$\begin{aligned} p_k(\alpha ):= \left( {\begin{array}{c}n\\ k\end{array}}\right) ^{-1} \sum _{1 \le i_1< \cdots <i_k \le n} \alpha _{i_1} \cdots \alpha _{i_k}, \quad \forall ~ \alpha = \left( \alpha _1, \ldots , \alpha _n\right) . \end{aligned}$$

We set $p_0 \left( \alpha \right) =1$ for convenience.

Throughout the paper, we fix an integer $m \in \left\{ 1, \ldots , n\right\}$ and set $F= p_{m}/ p_{m-1}$ and $\mathcal {F}= p_{\ell +1}/ p_{\ell }$, where ℓ=n-m. Note that both *F* and $\mathcal {F}$ are concave. Denote by $\Gamma ^+:= \left\{ \alpha \in \mathbb {R}^n;~\alpha _i>0, ~ i=1, \ldots , n\right\}$ the positive cone. The function $\mathcal {F}$ is called the inverse function of *F*, as it is easy to see for any $\alpha \in \Gamma ^+$ that

$$\begin{aligned} F \left( \alpha \right) =\frac{p_m \left( \alpha \right) }{p_{m-1} \left( \alpha \right) } =\frac{p_{n-m} \left( \alpha ^{-1}\right) / p_n \left( \alpha ^{-1}\right) }{p_{n-m+1} \left( \alpha ^{-1}\right) / p_n \left( \alpha ^{-1}\right) } = \frac{1}{\mathcal {F}\left( \alpha ^{-1}\right) }, \end{aligned}$$

that is,

2.2 $$\begin{aligned} F \left( \alpha \right) \mathcal {F}(\alpha ^{-1} ) = 1, \end{aligned}$$

where we set $\alpha ^{-1} = \left( \alpha _1^{-1}, \ldots , \alpha _n^{-1}\right)$.

### Hypersurfaces in Hyperbolic Space

In the following, we provide some common conventions and notations for the hyperboloid model of the hyperbolic space $\mathbb {H}^{n+1}$ in the Minkowski space $\mathbb {R}^{n+1,1}$ and for hypersurfaces in $\mathbb {H}^{n+1}$.

The Minkowski space $\mathbb {R}^{n+1,1}$ is an (n+2)-dimensional vector space equipped with the Lorentzian product $\langle \cdot , \cdot \rangle _1$:

$$ \langle X, Y \rangle _1:= \sum _{i=1}^{n+1} x_i y_i - x_{n+2} y_{n+2}, $$

where $X = \left( x_1,x_2, \ldots , x_{n+2}\right)$ and $Y = \left( y_1, y_2, \ldots , y_{n+2}\right)$. For convenience, we write $X= \left( x, x_{n+2}\right)$ and $Y = \left( y, y_{n+2}\right)$. We call a vector $X \in \mathbb {R}^{n+1,1}$ timelike if $\langle X, X \rangle _1<0$, lightlike if $\langle X,X \rangle _1 = 0$, and spacelike if $\langle X,X \rangle _1>0$. We call *X* future-directed if $x_{n+2}>0$ and past-directed if $x_{n+2}<0$. The hyperbolic space $\left( \mathbb {H}^{n+1}, \bar{g}\right)$ can be viewed as a hypersurface in $\left( \mathbb {R}^{n+1,1}, \langle \cdot , \cdot \rangle _1\right)$ equipped with the induced metric $\bar{g}$, known as the hyperboloid model:

$$ \mathbb {H}^{n+1} = \left\{ X \in \mathbb {R}^{n+1,1};~ \langle X,X \rangle _1= -1, \ x_{n+2} >0\right\} . $$

The isometry group of $\mathbb {H}^{n+1}$ is given by the positive Lorentz group $O_+ \left( n+1,1\right)$ consisting of (n+2)×(n+2) matrices $\mathcal {M}$ satisfying $\mathcal {M}^T J \mathcal {M} = J$ with $\mathcal {M} \left( \mathbb {H}^{n+1}\right) =\mathbb {H}^{n+1}$, where $J = \text {diag} \left( 1, \ldots ,1, -1\right)$. The geodesic distance between two points *X*, *Y* in $\mathbb {H}^{n+1}$ is denoted by $d_{\mathbb {H}^{n+1}} \left( X, Y\right)$, which can be given by

2.3 $$\begin{aligned} d_{\mathbb {H}^{n+1}} \left( X, Y\right) = \cosh ^{-1} \left( - \langle X, Y \rangle _1 \right) . \end{aligned}$$

In this paper, we set $\mathcal {O}= (0,1)$ as the origin of $\mathbb {H}^{n+1}$ and denote $r = d_{\mathbb {H}^{n+1}} \left( \mathcal {O}, \cdot \right)$ as the distance function from the origin. Here “0” stands for the zero vector in $\mathbb {R}^{n+1}$. As a timelike vector, we also denote $E_{n+2} = (0,1) \in \mathbb {R}^{n+1,1}$. Let $\left( \mathbb {S}^n, \sigma , D\right)$ denote the unit sphere equipped with the canonical metric σ and the Levi-Civita connection *D* in $\mathbb {R}^{n+1}$. For simplicity of notation, for any smooth function *f* on $\mathbb {S}^n$, we denote its covariant derivatives as $f_i:= D_i f$, $f_{ij}:= D_j D_i f$, etc. In addition, we always identify $\mathbb {S}^n$ as $\mathbb {S}^n \times \left\{ 0\right\}$ in $\mathbb {R}^{n+1,1}$.

For any $X \in \mathbb {H}^{n+1}$ with $X \ne \mathcal {O}$, there exist unique r>0 and $\theta \in \mathbb {S}^n$ such that

$$ X = \left( \lambda \left( r\right) \theta , \lambda ' \left( r\right) \right) , $$

where we set $\lambda \left( s\right) = \sinh s$. This gives a warped product structure of $\left( \mathbb {H}^{n+1}, \bar{g}\right)$ with $\bar{g} = dr^2+ \lambda \left( r\right) ^2 g_{\mathbb {S}^n}$. For convenience, we also write $\bar{g} = \langle \cdot , \cdot \rangle$.

Let Ω be a domain with smooth boundary $M = \partial \Omega$ in $\mathbb {H}^{n+1}$. Denote by *g* the induced metric and by ∇ the Levi-Civita connection on *M*, and denote by $\nu = \left( \hat{\nu }, \nu _{n+2}\right)$ the unit outer normal of *M* in $\mathbb {H}^{n+1}$. It is easy to see $\langle X, \nu \rangle _1=0$ and $\langle X-\nu , X-\nu \rangle _1=0$. Let $V = \lambda \left( r\right) \partial _r$ be a vector field in $\mathbb {H}^{n+1}$. Then in the hyperboloid model,

2.4 $$\begin{aligned} V = \lambda \left( r\right) \partial _r X = \left( \lambda \lambda ' \theta , \left( \lambda '\right) ^2-1\right) = \lambda ' X - E_{n+2}, \end{aligned}$$

which means $V \left( X\right)$ is the projection of $-E_{n+2}$ onto $T_X \mathbb {H}^{n+1}$. Define the support function $\tilde{u}$ of *M* as $\tilde{u}=\langle V, \nu \rangle$. Then

$$ \tilde{u} \left( X\right) = -\langle \nu , E_{n+2} \rangle _1 = \nu _{n+2} \left( X\right) , \quad X \in M. $$

Consequently, at any X∈M, the quantity $\left( \cosh r -\tilde{u}\right)$ is exactly the last coordinate of X-ν, i.e.,

2.5 $$\begin{aligned} \cosh r- \tilde{u} = - \langle X-\nu , E_{n+2} \rangle _1. \end{aligned}$$

Since $\cosh r-\tilde{u} \ge \lambda '-\lambda >0$, we see that X-ν is future lightlike.

Let $\left( h_{ij}\right)$ denote the second fundamental form of *M*, and let  denote the Weingarten matrix, where . In a local orthonormal frame, $h_{ij} = \langle \bar{\nabla }_{e_i} \nu , e_j \rangle$, and it holds $\nabla _k h_{ij}= \nabla _j h_{ik}$ by the Codazzi equation. Using (2.5) and (2.4), we see that2.6The principal curvatures $\kappa = \left( \kappa _1, \ldots , \kappa _n\right)$ of *M* are the eigenvalues of the Weingarten matrix. We call $\tilde{\kappa } = \left( \tilde{\kappa }_1, \ldots , \tilde{\kappa }_n\right)$ the shifted principal curvatures, where $\tilde{\kappa }_i = \kappa _i -1$. We say that Ω or its boundary $M =\partial \Omega$ is horo-convex (h-convex for short) if $\kappa _i \ge 1$ everywhere on *M* and is strictly h-convex if $\kappa _i >1$ on *M*.

The quermassintegrals $\left\{ W_k \right\} _{k=0}^n$ of a convex body Ω with smooth boundary $M = \partial \Omega$ in $\mathbb {H}^{n+1}$ are defined by (see [25]):

2.7 $$\begin{aligned} \begin{aligned}&W_0 \left( \Omega \right) = \text {Vol}\left( \Omega \right) , \quad W_1(\Omega ) = \left| M\right| /n,\\&W_{k+1} \left( \Omega \right) = \frac{1}{n-k} \int _{M} p_k \left( \kappa \right) \, d\mu -\frac{k}{n-k} W_{k-1} \left( \Omega \right) , \quad k=1, \ldots , n-1. \end{aligned} \end{aligned}$$

Andrews et al. [2] introduced the modified quermassintegrals $\{\widetilde{W}_k \}_{k=0}^n$ by setting

2.8 $$\begin{aligned} \widetilde{W}_k \left( \Omega \right) = \sum _{i=0}^k \left( -1\right) ^{k-i} C_k^i W_i \left( \Omega \right) , \quad k=0, \ldots , n. \end{aligned}$$

In particular, $\widetilde{W}_0(\Omega ) = \text {Vol}(\Omega )$ and $\widetilde{W}_1 (\Omega ) = \left| M\right| /n -\text {Vol}(\Omega )$. They proved that these modified quermassintegrals are monotone with respect to the inclusions of h-convex bounded domains. Define $\tilde{f}_k: \mathbb {R}_+ \rightarrow \mathbb {R}_+$ by $\tilde{f}_k \left( r\right) = \widetilde{W}_k \left( B(r)\right)$, where $B\left( r\right)$ is a geodesic ball of radius *r* in $\mathbb {H}^{n+1}$. We see that $\tilde{f}_k$ is positive and strictly increasing on (0,+∞).

### Horospherical Support Function

Every convex body in Euclidean space induces a function on the unit sphere, known as the support function, which is a powerful tool in the study of the geometry of convex bodies. In this subsection, we recall the properties of its counterpart for h-convex domains in hyperbolic space, called the horospherical support function. Readers can refer to [2, 17] for details.

The horospheres in $\mathbb {H}^{n+1}$ are complete hypersurfaces in hyperbolic space with constant principal curvatures equal to 1. The set of horospheres can be parameterized by $\mathbb {S}^n \times \mathbb {R}$:

$$ H_z \left( s\right) = \left\{ X \in \mathbb {H}^{n+1};~ -\langle X, \left( z,1\right) \rangle _1 = e^{s} \right\} , \quad \left( z,s\right) \in \mathbb {S}^n \times \mathbb {R}, $$

where *s* is the signed geodesic distance from the origin $\mathcal {O}$ to $H_z(s)$. The horo-ball $B_z \left( s\right)$ enclosed by the horosphere $H_z(s)$ is

$$ B_z \left( s\right) = \left\{ X \in \mathbb {H}^{n+1};~ -\langle X, \left( z,1\right) \rangle _1 < e^{s} \right\} . $$

It is clear from the geometric interpretation of *s* that the intersection of closed horo-balls $\overline{B_z(s_1)} \cap \overline{B_{-z}(s_2)}$ is nonempty if and only if $s_1+s_2 \ge 0$.

For any bounded domain Ω with boundary $M = \partial \Omega$ in $\mathbb {H}^{n+1}$, we define its horospherical support function $u_{\Omega }:\mathbb {S}^n \rightarrow \mathbb {R}$ by

2.9 $$\begin{aligned} u_{\Omega }(z) := \inf \left\{ s \in \mathbb {R};~\Omega \subset B_z(s)\right\} , \quad z \in \mathbb {S}^n. \end{aligned}$$

We always set $\varphi _{\Omega } = \exp \left( u_{\Omega }\right)$. If the domain is clear from the context, we will write $u_{\Omega }$ as *u* and $\varphi _{\Omega }$ as φ. It follows directly from (2.9) that $u_{\Omega } (z) + u_{\Omega } \left( -z\right) \ge 0$, $z \in \mathbb {S}^n$. This implies

2.10 $$\begin{aligned} \max _{\mathbb {S}^n} \varphi \cdot \min _{\mathbb {S}^n} \varphi \ge 1. \end{aligned}$$

Assume that *M* is strictly h-convex. Since X-ν is future lightlike, at any X∈M there exist unique $z \in \mathbb {S}^n$ and $u \in \mathbb {R}$ so that

2.11 $$\begin{aligned} X- \nu = e^{-u} \left( z,1\right) . \end{aligned}$$

We define the horospherical Gauss map $G: M \rightarrow \mathbb {S}^n$ as $G\left( X\right) = z$. It is known that *G* is a diffeomorphism, and for any $z \in \mathbb {S}^n$, the value *u* in (2.11) at $X = G^{-1} \left( z\right)$ is exactly $u_{\Omega } \left( z\right)$ defined in (2.9).

Recall that $\varphi := e^u >0$. Define a function $\psi : \mathbb {S}^n \rightarrow \mathbb {R}$ by

$$\begin{aligned} \psi = \frac{\left| D \varphi \right| ^2}{2 \varphi } + \frac{1}{2} \left( \varphi + \varphi ^{-1}\right) , \end{aligned}$$

and a symmetric 2-tensor A[φ] on $\mathbb {S}^n$ by

$$\begin{aligned} A [\varphi ] := D^2 \varphi - \frac{1}{2} \varphi ^{-1} \left| D \varphi \right| ^2 \sigma + \frac{1}{2} \left( \varphi - \varphi ^{-1}\right) \sigma . \end{aligned}$$

It is easy to see that

2.12 $$\begin{aligned} A[\varphi ] = D^2 \varphi + \varphi \sigma - \psi \sigma . \end{aligned}$$

In this paper, we occasionally abbreviate A[φ] as *A*.

The following formulas were proved in [17, Lemmas 2.2 and 3.9]; see also [2, Sect. 5].

#### Lemma 2.1

Let Ω be a bounded domain with smooth, strictly h-convex boundary $M= \partial \Omega$ in $\mathbb {H}^{n+1}$. Denote by *u* the horospherical support function of Ω, and set $\varphi =e^u$. For any $X \in \partial \Omega$ and $z \in \mathbb {S}^n$ so that $z= G(X) \in \mathbb {S}^n,$ we have2.132.142.15

It follows from (2.15) that A[φ]>0 whenever *M* is strictly h-convex. The converse is also true, meaning that given a positive function φ on $\mathbb {S}^n$ such that A[φ]>0, there exists a unique strictly h-convex hypersurface $M= \partial \Omega$ with $u_{\Omega } = \log \varphi$, see [2, Sect. 5.4].

Now, we can use the above Lemma 2.1 to express the speed function of the flow (1.2) via the horospherical support function.

#### Corollary 2.1

Let *M* be a smooth, strictly h-convex hypersurface in $\mathbb {H}^{n+1}$. Then for any $z \in \mathbb {S}^n,$ we have

2.16 $$\begin{aligned} \left( \frac{\lambda '-\tilde{u}}{F \left( \tilde{\kappa }\right) } - \tilde{u} \right) \left( X\right) = \mathcal {F}\left( A[\varphi \left( z\right) ]\right) - \psi \left( z\right) +\varphi \left( z\right) ^{-1}, \end{aligned}$$

where $X = G^{-1} \left( z\right) ,$ $F = p_m/ p_{m-1},$ and $\mathcal {F}= p_{\ell +1}/ p_{\ell }$ with ℓ=n-m.

#### Proof

It follows from (2.13) and (2.14) that $\left( \lambda '-\tilde{u}\right) \left( X\right) = \varphi \left( z\right) ^{-1}$ and $\tilde{u} \left( X\right) = \psi \left( z\right) - \varphi \left( z\right) ^{-1}$. Using (2.2), (2.15) and the 1-homogeneity of $\mathcal {F}$, we have

$$\begin{aligned} F \left( \tilde{\kappa }\right) ^{-1} = \mathcal {F}\left( \tilde{\kappa }^{-1}\right) = \mathcal {F}\left( \varphi A[\varphi ]\right) = \varphi \mathcal {F}\left( A[\varphi ]\right) . \end{aligned}$$

Putting the above formulas together, we obtain (2.16). □

In the following lemma, we provide formulas for φ, ψ and A[φ] when the domain Ω is a geodesic ball. These formulas will be used in Sect. 4 to study the behavior of solutions to the flow (1.2) when the initial hypersurface $M_0$ is a geodesic sphere.

#### Lemma 2.2

Let $\Omega = B (X, \rho )$ be a geodesic ball of radius ρ centered at $X = \left( x, x_{n+2}\right)$ in $\mathbb {H}^{n+1}$. Then

2.17 $$\begin{aligned} \varphi _{\Omega } \left( z\right) = e^{\rho } \left( x_{n+2} - \langle x,z \rangle \right) , \quad z \in \mathbb {S}^n. \end{aligned}$$

This implies

2.18 $$\begin{aligned} \varphi _{\Omega } A[\varphi _{\Omega }] = \frac{e^{2 \rho } -1}{2}\sigma ,\end{aligned}$$

2.19 $$\begin{aligned} \psi _{\Omega } = e^{\rho } x_{n+2} - \frac{e^{2 \rho }-1}{2\varphi _{\Omega }}. \end{aligned}$$

#### Proof

Formula (2.17) was proved in [17, Lemma 4.4]. Formula (2.18) follows from (2.15) and the fact that the principal curvatures of a geodesic sphere of radius ρ in $\mathbb {H}^{n+1}$ are all equal to $\cosh \left( \rho \right) / \sinh \left( \rho \right)$. For the Formula (2.19), we note that $\left| D \varphi _{\Omega }\right| ^2 = e^{2 \rho } \left( \left| x\right| ^2-\langle x, z \rangle ^2\right)$. Then

$$\begin{aligned} 2 \varphi _{\Omega } \psi _{\Omega }&= \left| D \varphi _{\Omega }\right| ^2+ \varphi _{\Omega }^2+ 1 \\&= e^{2 \rho } \left( \left| x\right| ^2-\langle x,z \rangle ^2+ x_{n+2}^2-2 x_{n+2} \langle x, z \rangle + \langle x,z \rangle ^2\right) +1\\&= e^{2\rho } \left( 2x_{n+2}^2-2x_{n+2} \langle x,z \rangle \right) -e^{2\rho }+1\\&= 2 e^{\rho } x_{n+2} \varphi _{\Omega } -e^{2\rho } +1. \end{aligned}$$

Dividing both sides of the above formula by $2 \varphi _{\Omega }$ yields (2.19). □

To conclude this section, we derive two formulas for calculating the evolution equations in the next Sect. 3.

#### Lemma 2.3

In a local orthonormal frame, we have

2.20 $$\begin{aligned} D_j D_i \psi&= \varphi ^{-1} \left( \langle D\varphi , D\psi \rangle \delta _{ij} - \psi _i \varphi _j - \psi _j \varphi _i\right) + \varphi ^{-1} \langle D \varphi , D A_{ij} \rangle \nonumber \\&\quad + \varphi ^{-1} \left( A^2\right) _{ij} + \left( \varphi ^{-1} \psi - 1\right) A_{ij}, \end{aligned}$$

2.21 $$\begin{aligned} D_j D_i \left( \varphi \psi \right)&= \langle D \varphi , D A_{ij} \rangle + \left( A^2\right) _{ij} + \left( 2 \psi -\varphi \right) A_{ij} + \langle D\varphi , D \psi \rangle \delta _{ij} \nonumber \\&\quad + \psi \left( \psi -\varphi \right) \delta _{ij}. \end{aligned}$$

#### Proof

First, it follows from the Ricci identity that $D^3 \varphi + D\varphi \otimes \sigma$ is a fully symmetric 3-tensor on $\mathbb {S}^n$. Then by (2.12),

2.22 $$\begin{aligned} D_k A_{ij}+ D_k \psi \delta _{ij} = D_j A_{ik}+ D_j \psi \delta _{ik}. \end{aligned}$$

By a direct calculation,

$$\begin{aligned} D_i \psi = \varphi ^{-1} \varphi _k \varphi _{ki} - \frac{1}{2} \varphi ^{-2} \left| D \varphi \right| ^2 \varphi _i+ \frac{1}{2} \varphi _i-\frac{1}{2} \varphi ^{-2} \varphi _i= \varphi ^{-1} \varphi _k A_{ki}. \end{aligned}$$

Differentiating the above formula and using (2.22), we have

$$\begin{aligned} D_jD_i \psi&= -\varphi ^{-2} \varphi _j \varphi _k A_{ki} + \varphi ^{-1} \varphi _{kj} A_{ki}+ \varphi ^{-1} \varphi _k A_{ki,j}\\&=- \varphi ^{-1} \varphi _j \psi _i+ \varphi ^{-1} \left( A_{kj}+ \psi \delta _{kj} - \varphi \delta _{kj}\right) A_{ki} \\&\quad +\varphi ^{-1} \varphi _k \left( A_{ij,k} + \psi _k \delta _{ij} -\psi _j \delta _{ik}\right) \\&= \varphi ^{-1} \left( \varphi _k \psi _k \delta _{ij} - \varphi _i \psi _j-\varphi _j \psi _i\right) + \varphi ^{-1} \varphi _k A_{ij,k}\\&\quad + \varphi ^{-1} A_{ik} A_{kj} + \left( \varphi ^{-1}\psi -1\right) A_{ij}. \end{aligned}$$

Thus, we obtain (2.20). Note that

$$\begin{aligned} D_j D_i \left( \varphi \psi \right)&= \varphi \psi _{ij} + \varphi _i \psi _j + \varphi _j \psi _i+ \psi \varphi _{ij}\\&= \varphi \psi _{ij} + \varphi _i \psi _j+ \varphi _j \psi _i+\psi \left( A_{ij} + \psi \delta _{ij} - \varphi \delta _{ij}\right) . \end{aligned}$$

Then Formula (2.21) follows by substituting (2.20) into the above formula. □

## Evolution Equations

Along a general flow $\partial _t X = \eta \nu$ for strictly h-convex hypersurfaces $\left\{ M_t\right\}$ in $\mathbb {H}^{n+1}$, it was shown in [2, Eq. (5.13)] (see also [17, Eq. (5.3)]) that the scalar evolution equation of the horospherical support function $u \left( \cdot , t\right)$ of $\Omega _t$ is given by

$$\begin{aligned} \frac{\partial }{\partial t} u = \eta \circ G_t^{-1}, \end{aligned}$$

where $G_t: M_t \rightarrow \mathbb {S}^n$ is the horospherical Gauss map of $\Omega _t$. Recall our notation that $\varphi = \exp \left( u\right)$. Then we have from (2.16) that the scalar equation associated with the flow (1.2) is

3.1 $$\begin{aligned} \left\{ \begin{aligned}&\frac{\partial }{\partial t} \varphi = \varphi \mathcal {F}(A) - \varphi \psi +1,\\&\varphi \left( \cdot , 0\right) = \varphi _0 \left( \cdot \right) , \end{aligned} \right. \end{aligned}$$

where $\mathcal {F}(A) =p_{\ell +1} \left( A\right) / p_{\ell } \left( A\right)$ with ℓ=n-m, and $\varphi _0= \exp \left( u_{\Omega _0}\right)$. We say a solution $\varphi : \mathbb {S}^n \times [0, T)$ to the Eq. (3.1) is strictly h-convex if A[φ]>0 on $\mathbb {S}^n \times [0, T)$. Equivalently, the function $\log \varphi \left( \cdot , t\right)$ is the horospherical support function of a strictly h-convex hypersurface $M_t$ that satisfies the flow (1.2).

Unlike the scalar formulation in [14], which relies on representing $M_t$ as a radial graph over $\mathbb {S}^n$ and therefore requires $M_t$ to enclose the origin, the scalar equation (3.1) based on the horospherical support function remains valid as long as the evolving hypersurface is strictly h-convex. In particular, it does not depend on the relative position of the hypersurface with respect to the origin. For example, it will be used in Sect. 4 to obtain an explicit solution to the flow (1.2) when the initial domain $\Omega _0$ is an arbitrary geodesic ball.

We denote the associated linearized parabolic operator of Eq. (3.1) as

$$\begin{aligned} \mathcal {L}:= \frac{\partial }{\partial t} - \varphi \dot{\mathcal {F}}^{ij} D_j D_i. \end{aligned}$$

In the following calculation, we will use the 1-homogeneity of $\mathcal {F}\left( A\right)$, that is,

3.2 $$\begin{aligned} \mathcal {F}\left( A\right) = \dot{\mathcal {F}}^{ij} A_{ij}. \end{aligned}$$

Moreover, for convenience, we will work in normal coordinates around the point we calculate so that $\sigma _{ij} = \delta _{ij}$ at that point.

### Lemma 3.1

Along the Eq. (3.1), we have

3.3 $$\begin{aligned} \mathcal {L}\psi&= \dot{\mathcal {F}}^{ij} \left( 2 \varphi _i \psi _j -\langle D \varphi , D \psi \rangle \delta _{ij} \right) - \langle D \varphi , D \psi \rangle -\dot{\mathcal {F}}^{ij} \left( A^2\right) _{ij} + \left( \varphi - \varphi ^{-1}\right) \mathcal {F}- \psi ^2+1. \end{aligned}$$

### Proof

By a direct calculation,

3.4 $$\begin{aligned} \frac{\partial }{\partial t} \psi&= \varphi ^{-1} \langle D \varphi , D \partial _t \varphi \rangle - \varphi ^{-1} \left( \psi -\varphi \right) \partial _t \varphi \nonumber \\&= \varphi ^{-1} \langle D \varphi , D \left( \varphi \left( \mathcal {F}-\psi \right) \right) \rangle -\left( \psi -\varphi \right) \left( \mathcal {F}-\psi +\varphi ^{-1}\right) \nonumber \\&= \varphi ^{-1} \left| D \varphi \right| ^2 \left( \mathcal {F}-\psi \right) + \langle D \varphi , D\left( \mathcal {F}-\psi \right) \rangle - \left( \psi -\varphi \right) \mathcal {F}+ \left( \psi -\varphi \right) \left( \psi - \varphi ^{-1}\right) \nonumber \\&= \langle D \varphi , D \mathcal {F} \rangle - \langle D\varphi , D \psi \rangle + \left( \psi - \varphi ^{-1}\right) \mathcal {F}-\psi ^2+1. \end{aligned}$$

On the other hand, using (2.20) and (3.2), we have

$$\begin{aligned} \varphi \dot{\mathcal {F}}^{ij} D_j D_i \psi&= \dot{\mathcal {F}}^{ij} \left( \langle D \varphi , D\psi \rangle \delta _{ij} -2 \varphi _i \psi _j \right) \\ + \langle D \varphi , D\mathcal {F} \rangle + \mathcal {F}^{ij} \left( A^2\right) _{ij} + \left( \psi -\varphi \right) \mathcal {F}. \end{aligned}$$

Combining the above formula with (3.4), we obtain (3.3). □

### Lemma 3.2

Along the Eq. (3.1), we have

3.5 $$\begin{aligned} \frac{\partial }{\partial t} A_{ij}&= \varphi \mathcal {F}_{ij}- \langle D \varphi , D \mathcal {F} \rangle \delta _{ij} + \varphi _i \mathcal {F}_j + \varphi _j \mathcal {F}_i - \langle D \varphi , D A_{ij} \rangle \nonumber \\&\quad - \left( A^2\right) _{ij}+ \mathcal {F}A_{ij} - \left( 2 \psi -\varphi \right) A_{ij} +\varphi ^{-1} \mathcal {F}\delta _{ij}, \end{aligned}$$

3.6 $$\begin{aligned} \mathcal {L}\mathcal {F}&= \dot{\mathcal {F}}^{ij} \left( 2 \varphi _i \mathcal {F}_j- \langle D \varphi , D\mathcal {F} \rangle \delta _{ij}\right) - \langle D \varphi , D\mathcal {F} \rangle \nonumber \\&\quad -\dot{\mathcal {F}}^{ij} \left( A^2\right) _{ij} + \mathcal {F}^2- \left( 2 \psi - \varphi -\varphi ^{-1} \dot{\mathcal {F}}^{ij} \delta _{ij}\right) \mathcal {F}. \end{aligned}$$

### Proof

Using Formula (2.12) and the Eq. (3.1), we have

3.7 $$\begin{aligned} \frac{\partial }{\partial t} A_{ij}&= \partial _t \left( \varphi _{ij} + \left( \varphi -\psi \right) \delta _{ij} \right) \nonumber \\&= \left( \varphi \mathcal {F}- \varphi \psi +1\right) _{ij} + \partial _t \left( \varphi - \psi \right) \delta _{ij}. \end{aligned}$$

For the first term on the right-hand side of (3.7), using (2.21), we have

$$\begin{aligned} \left( \varphi \mathcal {F}- \varphi \psi +1\right) _{ij}&= \varphi _{ij} \mathcal {F}+ \varphi _i \mathcal {F}_j+ \varphi _j \mathcal {F}_i +\varphi \mathcal {F}_{ij} - \left( \varphi \psi \right) _{ij}\\&= A_{ij} \mathcal {F}+ \left( \psi -\varphi \right) \mathcal {F}\delta _{ij} + \varphi _i \mathcal {F}_j+ \varphi _j \mathcal {F}_i + \varphi \mathcal {F}_{ij} - \langle D \varphi , D A_{ij} \rangle \\&\quad - \left( A^2\right) _{ij} - \left( 2\psi - \varphi \right) A_{ij} - \langle D \varphi , D\psi \rangle \delta _{ij} - \psi \left( \psi - \varphi \right) \delta _{ij}. \end{aligned}$$

On the other hand, using (3.4), we have

$$\begin{aligned} \partial _t \left( \varphi - \psi \right)&= \varphi \mathcal {F}- \varphi \psi +1 - \langle D \varphi , D\mathcal {F} \rangle + \langle D \varphi , D \psi \rangle -\left( \psi -\varphi ^{-1}\right) \mathcal {F}+ \psi ^2-1\\&= - \langle D \varphi , D\mathcal {F} \rangle + \langle D \varphi , D \psi \rangle - \left( \psi - \varphi -\varphi ^{-1}\right) \mathcal {F}+ \psi \left( \psi - \varphi \right) . \end{aligned}$$

Substituting the above two formulas into (3.7), we obtain (3.5). Since $\mathcal {F}= \dot{\mathcal {F}}^{ij} A_{ij}$ and $\partial _t \mathcal {F}= \dot{\mathcal {F}}^{ij} \partial _t A_{ij}$, the Eq. (3.6) follows by contracting both sides of (3.5) with $\dot{\mathcal {F}}^{ij}$. □

The following evolution equations of the metric $g_{ij}$ and the shifted second fundamental form $\tilde{h}_{ij}$ of $M_t$ were proved in [14, Eqs. (3.6) and (3.7)]. Here, we abbreviate $F \left( \tilde{\kappa }\right)$ as *F*.

### Lemma 3.3

Along the flow (1.2), we have

3.8 $$\begin{aligned} \frac{\partial }{\partial t} g_{ij}&= 2 \left( \frac{\lambda '-\tilde{u}}{F} - \tilde{u} \right) h_{ij}, \end{aligned}$$

3.9 $$\begin{aligned} \frac{\partial }{\partial t} \tilde{h}_{ij}&= \frac{\lambda '-\tilde{u}}{F^2} \dot{F}^{kl} \nabla _l \nabla _k \tilde{h}_{ij} + \frac{\lambda '- \tilde{u}}{F^2} \ddot{F}^{kl, pq} \nabla _i \tilde{h}_{kl} \nabla _j \tilde{h}_{pq} \nonumber \\&\quad + \frac{1}{F} \left( \nabla _j F \nabla _i \frac{\lambda '-\tilde{u}}{F}+ \nabla _i F \nabla _j \frac{\lambda '-\tilde{u}}{F} \right) + \frac{1+F}{F} \langle V, \nabla \tilde{h}_{ij} \rangle \nonumber \\&\quad + \left( \frac{\lambda '-\tilde{u}}{F^2} \left( \dot{F}^{kl} (\tilde{h}^2)_{kl}+ F\right) + \lambda '-2 \tilde{u} \right) \tilde{h}_{ij} \nonumber \\&\quad - \left( \frac{\lambda '-\tilde{u}}{F^2} \dot{F}^{kl} g_{kl} + \frac{\tilde{u}}{F} + 2\tilde{u} \right) (\tilde{h}^2)_{ij} + \left( \lambda '-\tilde{u}\right) \left( 1+\frac{\dot{F}^{kl} (\tilde{h}^2)_{kl} }{F^2} \right) g_{ij}. \end{aligned}$$

## Evolution of Geodesic Balls

In this section, we prove that if the initial domain $\Omega _0$ is a geodesic ball in $\mathbb {H}^{n+1}$, then $\Omega _t$ is a geodesic ball with the same radius for any t>0 along the flow (1.2). Moreover, we show that $\Omega _t$ converges to a geodesic ball centered at the origin exponentially fast as $t \rightarrow \infty$ by providing the following explicit solution to the flow (1.2).

### Proposition 4.1

Let ρ>0 and $Y_0 \in \mathbb {H}^{n+1},$ and let $d_0 = d_{\mathbb {H}^{n+1}} \left( \mathcal {O}, Y_0\right)$. For any t>0, we let *Y*(*t*) be a point on the geodesic connecting $\mathcal {O}$ and $Y_0$ such that the distance $d(t) = d_{\mathbb {H}^{n+1}} \left( \mathcal {O}, Y(t)\right)$ is given by

4.1 $$\begin{aligned} \tanh \frac{d(t)}{2} = \tanh \frac{d_0}{2} \exp \left( - e^{\rho } t\right) . \end{aligned}$$

If $\Omega _0= B \left( Y_0, \rho \right)$ in the flow (1.2), then $\Omega _t = B \left( Y(t), \rho \right)$. Indeed, *Y*(*t*) is the solution to the following equation:

4.2 $$\begin{aligned} {\left\{ \begin{array}{ll} Y'(t) = e^{\rho } E_{n+2} + e^{\rho } \langle Y(t), E_{n+2} \rangle _1 Y(t), \quad t \ge 0,\\ Y(0) = Y_0. \end{array}\right. } \end{aligned}$$

### Proof

Let *d*(*t*) be a function defined by (4.1). Differentiating both sides of (4.1), we have

$$\begin{aligned} d'(t) = - e^{\rho } \lambda \left( d (t)\right) . \end{aligned}$$

Let $\theta \in \mathbb {S}^n$ such that $Y_0 = \left( \lambda \left( d_0\right) \theta , \lambda ' \left( d_0\right) \right)$, and set $Y(t) = \left( \lambda \left( d(t)\right) \theta , \lambda ' \left( d(t)\right) \right)$. For simplicity of notation, we write $\lambda = \lambda \left( d(t)\right)$ and $\lambda '= \lambda ' \left( d(t)\right)$ in the rest of the proof. It is straightforward to check that *Y*(*t*) is the solution to the Eq. (4.2):

$$\begin{aligned} Y' \left( t\right) = \left( \lambda ' \theta , \lambda \right) d' \left( t\right) = -e^{\rho } \left( \lambda ' \lambda \theta , \lambda ^2\right) = -e^{\rho } (\lambda ' \lambda \theta , \left( \lambda '\right) ^2-1)= - e^{\rho }\lambda ' Y \left( t\right) + e^{\rho } E_{n+2}. \end{aligned}$$

Next, we prove that $\Omega _t = B \left( Y(t), \rho \right)$ is the solution to the flow (1.2). By (2.17), we have

$$\begin{aligned} \varphi _{\Omega _t} \left( z\right) = e^{\rho } \left( \lambda ' \left( d(t)\right) - \lambda \left( d(t)\right) \langle \theta , z \rangle \right) . \end{aligned}$$

Then

4.3 $$\begin{aligned} \frac{\partial }{\partial t} \varphi _{\Omega _t} \left( z\right)&= e^{\rho } \left( \lambda - \lambda ' \langle \theta , z \rangle \right) d'(t)= -e^{2 \rho } \left( \left( \lambda '\right) ^2- \lambda \lambda ' \langle \theta , z \rangle -1 \right) = -e^{\rho } \lambda ' \varphi _{\Omega _t} \left( z\right) + e^{2 \rho }. \end{aligned}$$

On the other hand, using (2.18) and (2.19), we have for $\Omega _t$,

4.4 $$\begin{aligned} \varphi \mathcal {F}\left( A\right) - \varphi \psi +1 = \frac{e^{2 \rho } -1}{2} - \left( \varphi e^{\rho } \lambda '- \frac{e^{2 \rho } -1}{2}\right) +1 = e^{2 \rho } - e^{\rho } \lambda ' \varphi . \end{aligned}$$

Comparing (4.3) with (4.4), we conclude that the function $\varphi _{\Omega _t} \left( z\right)$ is the solution to (3.1), and thus $\Omega _t$ is the solution to the flow (1.2). This completes the proof. □

## A Priori Estimates

Throughout this section, we assume that $\varphi : \mathbb {S}^n \times [0, T)$ is a strictly h-convex solution to the scalar equation (3.1); and the corresponding embedding $X: M \times [0, T) \rightarrow \mathbb {H}^{n+1}$ is a strictly h-convex solution to the flow (1.2). In what follows, *C* denotes a positive constant depending only on the initial hypersurface $M_0$ (but not on *T*), which may vary from line to line.

### $C^0$ and $C^1$ Estimates

#### Lemma 5.1

We have

5.1 $$\begin{aligned} C^{-1}< \varphi < C \quad \text {on}~\mathbb {S}^n \times [0, T), \end{aligned}$$

where *C* is a positive constant depending only on $M_0$.

#### Proof

Denote $\varphi _{\max } \left( t\right) = \max _{z \in \mathbb {S}^n} \varphi \left( z, t\right)$. At the spatial maximum point of $\varphi \left( \cdot , t\right)$, we have Dφ=0 and $D^2 \varphi \le 0$. It follows that

$$ \psi = \left( \varphi + \varphi ^{-1}\right) /2, \quad 0 < A[\varphi ] \le \left( \varphi -\varphi ^{-1}\right) \sigma /2 $$

at the point. Hence, by the monotonicity and 1-homogeneity of $\mathcal {F}$,

$$ \mathcal {F}\left( A\right) \le \mathcal {F}\left( \left( \varphi - \varphi ^{-1}\right) \sigma / 2\right) = \left( \varphi - \varphi ^{-1}\right) / 2. $$

Substituting the above estimates into the Eq. (3.1), we obtain $\partial _t \varphi \le 0$ at the point. Hence $\left( \varphi _{\max }\right) '\left( t\right) \le 0$ and then $\varphi _{\max } \left( t\right) \le \max _{z \in \mathbb {S}^n} \varphi \left( z, 0\right)$. The lower bound of φ follows by (2.10) and its upper bound. This completes the proof. □

#### Lemma 5.2

We have

5.2 $$\begin{aligned} \left| D \varphi \right| < C \quad \text {on}~\mathbb {S}^n \times [0, T), \end{aligned}$$

where *C* is a positive constant depending only on $M_0$.

#### Proof

By Lemma 5.1, we let $C_0$ be a constant depending only on $M_0$ so that $C_0^{-1}<\varphi <C_0$ on $\mathbb {S}^n \times [0, T)$. It follows from (2.17) that $\Omega _t$ lies inside a geodesic ball of radius $\log C_0$ centered at the origin. Then by (2.13), we have $\psi \le \cosh \left( \log C_0\right)$, i.e.,

$$\begin{aligned} \frac{\left| D \varphi \right| ^2}{2\varphi } + \frac{1}{2}\left( \varphi + \varphi ^{-1}\right) \le \frac{1}{2} \left( C_0+C_0^{-1}\right) . \end{aligned}$$

Then the gradient estimate (5.2) follows by applying $\varphi > C_0^{-1}$ to the above estimate. □

### Estimates of $\mathcal {F}$

For the estimates in this subsection, we will use the following properties of the function $\mathcal {F}= p_{\ell +1}/ p_{\ell }$:

5.3 $$\begin{aligned}&\dot{\mathcal {F}}^{ij} \delta _{ij} \in [1, \ell +1], \end{aligned}$$

5.4 $$\begin{aligned}&\dot{\mathcal {F}}^{ij} \left( A^2\right) _{ij} \in [\mathcal {F}^2, \left( n-\ell \right) \mathcal {F}^2], \end{aligned}$$

where *A* is a positive symmetric matrix; see e.g. [14, Corollary 2.3].

#### Lemma 5.3

We have

5.5 $$\begin{aligned} \mathcal {F}\left( A\right) \le C \quad \text {on}~ \mathbb {S}^n \times [0, T), \end{aligned}$$

where *C* is a positive constant depending only on $M_0$.

#### Proof

Let $Q = \mathcal {F}\left( A\right) + \psi$. It follows from (5.1) and (5.2) that φ, $\varphi ^{-1}$ and ψ have a uniform upper bound on $\mathbb {S}^n \times [0, T)$. Then by the Eqs. (3.6), (3.3) and the property (5.3), there exists a constant *C* depending only on $M_0$ such that

$$\begin{aligned} \mathcal {L}\mathcal {F}&\le \dot{\mathcal {F}}^{ij} \left( 2 \varphi _i \mathcal {F}_j - \langle D \varphi , D\mathcal {F} \rangle \delta _{ij}\right) - \langle D \varphi , D \mathcal {F} \rangle - \dot{\mathcal {F}}^{ij} \left( A^2\right) _{ij}+ \mathcal {F}^2 + C \mathcal {F},\\ \mathcal {L}\psi&\le \dot{\mathcal {F}}^{ij} \left( 2 \varphi _i \psi _j -\langle D \varphi , D \psi \rangle \delta _{ij}\right) - \langle D \varphi , D \psi \rangle -\dot{\mathcal {F}}^{ij} \left( A^2\right) _{ij} + C\mathcal {F}+C. \end{aligned}$$

By adding the above two estimates and using (5.4), we obtain

$$\begin{aligned} \mathcal {L}Q \le \dot{\mathcal {F}}^{ij} \left( 2 \varphi _i Q_j- \langle D \varphi , D Q \rangle \delta _{ij}\right) - \mathcal {F}^2+ C \mathcal {F}+C. \end{aligned}$$

Since ψ is uniformly bounded, the right-hand side is negative for sufficiently large *Q*, which implies that *Q* is bounded from above by the maximum principle. Hence $\mathcal {F}$ is uniformly bounded from above. □

### $C^2$ Estimates

Recall (2.2), which states that $\mathcal {F}\left( \alpha \right) F (\alpha ^{-1}) =1$ for any $\alpha \in \Gamma ^+$. Then by (2.15), we have $F \left( \tilde{\kappa }\right) = \mathcal {F}\left( \varphi A\right) ^{-1} = \varphi ^{-1} \mathcal {F}\left( A\right) ^{-1}$. On the other hand, the Formula (2.14) states $\left( \lambda '-\tilde{u}\right) \circ G^{-1} = \varphi ^{-1}$. Thus, Lemmas 5.1 and 5.3 imply the following estimates.

#### Lemma 5.4

Along the flow (1.2), we have

5.6 $$\begin{aligned}&C^{-1}< \lambda ' -\tilde{u}< C, \quad \left| \tilde{u}\right|<C, \quad r <C, \end{aligned}$$

5.7 $$\begin{aligned}&F \left( \tilde{\kappa }\right) > C^{-1} , \end{aligned}$$

where *C* is a positive constant that depends only on $M_0$.

The following tensor maximum principle was proved by Andrews [1, Theorem 3.2].

#### Theorem 5.1

([1]) Let $S_{ij}$ be a smooth time-varying symmetric tensor field on a compact manifold *M*,  satisfying

$$\begin{aligned} \frac{\partial }{\partial t} S_{ij} =a^{kl} \nabla _k \nabla _l S_{ij} + b^k \nabla _k S_{ij} +N_{ij}, \end{aligned}$$

where $a^{kl}$ and $b^k$ are smooth, ∇ is a (possibly time-dependent) smooth symmetric connection, and $a^{kl}$ is positive definite everywhere. Suppose thatwhenever $S_{ij} \ge 0$ and $S_{ij} v^j=0,$ where the supremum is taken over all n×n matrix Γ. If $S_{ij}$ is positive definite everywhere on *M* at t=0 and on ∂M for $0 \le t \le T,$ then it is positive on M×[0,T].

It was shown in [14, Theorem 4.3] that the evolving hypersurface $M_t$ is strictly h-convex if the initial hypersurface $M_0$ is h-convex. In the following lemma, we will show that the shifted principal curvatures of $M_t$ have a uniform positive lower bound if $M_0$ is strictly h-convex.

#### Lemma 5.5

We have

5.8 $$\begin{aligned} \tilde{\kappa }_i \ge C^{-1}, \quad i=1, \ldots , n, \end{aligned}$$

where *C* is a positive constant depending only on $M_0$.

#### Proof

Let $S_{ij} = \tilde{h}_{ij} - \varepsilon g_{ij}$, where ε is a positive constant determined later. For now, we can just assume that ε satisfies $S_{ij} >0$ on $M_0$. Note here that ∇S is a fully symmetric 3-tensor.

It follows from (3.8) and (3.9) that the evolution equation of $S_{ij}$ is given by

$$\begin{aligned} \frac{\partial }{\partial t} S_{ij}&= \frac{\lambda '-\tilde{u}}{F^2} \dot{F}^{kl} \nabla _l \nabla _k S_{ij} + \frac{\lambda '- \tilde{u}}{F^2} \ddot{F}^{kl, pq} \nabla _i \tilde{h}_{kl} \nabla _j \tilde{h}_{pq} \\&\quad + \frac{1}{F} \left( \nabla _j F \nabla _i \frac{\lambda '-\tilde{u}}{F}+ \nabla _i F \nabla _j \frac{\lambda '-\tilde{u}}{F} \right) + \frac{1+F}{F} \langle V, \nabla S_{ij} \rangle \\&\quad + \left( \frac{\lambda '-\tilde{u}}{F^2} \left( \dot{F}^{kl} (\tilde{h}^2)_{kl}+ F\right) + \lambda '-2 \tilde{u} \right) \left( S_{ij}+ \varepsilon g_{ij}\right) \\&\quad - \left( \frac{\lambda '-\tilde{u}}{F^2} \dot{F}^{kl} g_{kl} + \frac{\tilde{u}}{F} + 2\tilde{u} \right) (\left( S+\varepsilon g\right) ^2)_{ij} \\&\quad + \left( \lambda '-\tilde{u}\right) \left( 1+\frac{\dot{F}^{kl} (\tilde{h}^2)_{kl} }{F^2} \right) g_{ij}\\&\quad - 2 \varepsilon \left( \frac{\lambda '-\tilde{u}}{F} - \tilde{u} \right) \left( S_{ij} + \left( 1+\varepsilon \right) g_{ij}\right) . \end{aligned}$$

Let us prove that $S_{ij}>0$ on M×[0,T) for ε>0 small enough. To apply the maximum principle in Theorem 5.1, we assume that $S_{ij} \ge 0$ on $M_{t_0}$ and $S_{ij} v^j=0$ at $\left( x_0, t_0\right)$ for some unit vector $v \in T_{x_0} M_{t_0}$, where $t_0 \in (0, T)$. By continuity, we can also assume that $\tilde{\kappa }_1< \tilde{\kappa }_2< \cdots <\tilde{\kappa }_n$ at $\left( x_0, t_0\right)$. Then we choose a normal coordinate of $M_{t_0}$ around $\left( x_0, t_0\right)$ so that $v=e_1$, $S_{11} = \tilde{\kappa }_1 - \varepsilon =0$, $S_{kk} = \tilde{\kappa }_k -\varepsilon >0$ ($2 \le k \le n$), and then $\nabla S_{11}=0$ at $\left( x_0, t_0\right)$. It is sufficient to show that5.9is nonnegative at $\left( x_0, t_0\right)$ provided ε is sufficiently small. Note that, by taking , at $\left( x_0, t_0\right)$ we haveOn the other hand, due to the concavity of $\mathcal {F}$, a similar argument as in [4, Sect. 3] implies

$$\begin{aligned} \ddot{F}^{kl, pq} \nabla _1 \tilde{h}_{kl} \nabla _1 \tilde{h}_{pq} \ge 2 \frac{\left| \nabla _1 F\right| ^2}{F} - 2 \sum _{k=2}^n \dot{F}^k \frac{(\nabla _1\tilde{h}_{kk} )^2 }{\tilde{\kappa }_k}. \end{aligned}$$

It follows from the above two estimates that5.10where we used the Cauchy–Schwarz inequality in the last line. Similar to (5.4), we have $\sum _{k=2}^n \dot{F}^k \tilde{\kappa }_k \left( \tilde{\kappa }_k-\varepsilon \right) \le \sum _{k=1}^n \dot{F}^k \tilde{\kappa }_k^2 \le (n-m+1) F^2$. On the other hand, we have

$$ \sum _{s=2}^n \dot{F}^s \left| \nabla _1\tilde{h}_{ss}\right| \ge \left| \sum _{s=2}^n \dot{F}^s \nabla _1\tilde{h}_{ss}\right| = \left| \nabla _1 F\right| . $$

Putting these estimates into the right-hand side of (5.10), we arrive atNow, we treat the second term on the right-hand side of (5.9). Using (2.6), we have

$$\begin{aligned} \frac{2}{F} \nabla _1 F \nabla _1 \frac{\lambda '-\tilde{u}}{F}&= -\frac{2}{F^2} \left( \nabla _1 F \right) \tilde{\kappa }_1 \langle V, e_1 \rangle -2\frac{\lambda '-\tilde{u}}{F^3} \left| \nabla _1 F\right| ^2\\&= -\frac{2\varepsilon }{F^2} \left( \nabla _1 F\right) \langle V, e_1 \rangle -2\frac{\lambda '-\tilde{u}}{F^3} \left| \nabla _1 F\right| ^2\\&\ge - \frac{2\varepsilon }{n-m+1} \frac{\lambda '-\tilde{u}}{F^4} \left| \nabla _1 F\right| ^2\\&\quad - 2\frac{\lambda '-\tilde{u}}{F^3} \left| \nabla _1 F\right| ^2 - \frac{n-m+1}{2} \frac{\langle V, e_1 \rangle ^2}{\lambda '-\tilde{u}} \varepsilon . \end{aligned}$$

Putting the above two estimates into (5.9), we have

5.11 $$\begin{aligned} Q_1&\ge -\frac{n-m+1}{2} \frac{\langle V, e_1 \rangle ^2}{\lambda '-\tilde{u}} \varepsilon + \left( \left( \lambda '-\tilde{u}\right) \left( F^{-2}\dot{F}^{kl} (\tilde{h}^2)_{kl}+ F^{-1}\right) + \lambda '-2 \tilde{u} \right) \varepsilon \nonumber \\&\quad - \left( \frac{\lambda '-\tilde{u}}{F^2} \dot{F}^{kl} g_{kl} + \frac{\tilde{u}}{F} + 2\tilde{u} \right) \varepsilon ^2- 2 \left( \frac{\lambda '-\tilde{u}}{F} -\tilde{u} \right) \left( \varepsilon ^2+\varepsilon \right) \nonumber \\&\quad +\left( \lambda '-\tilde{u}\right) \left( 1+\frac{\dot{F}^{kl} (\tilde{h}^2)_{kl} }{F^2} \right) . \end{aligned}$$

Note that, similar to (5.3) and (5.4), we have $\dot{F}^{kl}g_{kl} \in [1,m]$ and $F^{-2} \dot{F}^{kl} (\tilde{h}^2)_{kl} \in [1,n-m+1]$. Then the uniform lower bound of *F* in (5.7) and the $C^0$-estimate in (5.6) imply that the absolute values of the coefficients of ε and $\varepsilon ^2$ are bounded from above by a uniform constant *C*, and the last term on the right-hand side of (5.11) has a uniform lower bound:

$$\begin{aligned} \left( \lambda '-\tilde{u}\right) \left( 1+\frac{\dot{F}^{kl} (\tilde{h}^2)_{kl} }{F^2} \right) \ge \lambda '-\tilde{u} \ge C^{-1}. \end{aligned}$$

Consequently, there exists a constant $C_0$ depending only on $M_0$ so that for any $\varepsilon <C_0^{-1}$, it holds $Q_1 >0$ at $\left( x_0, t_0\right)$. Finally, we can take $\varepsilon = \min \left\{ \min _{M_0} \tilde{\kappa }_i, C_0^{-1}\right\} /2$ in the definition of $S_{ij}$ so that $\left( S_{ij}\right) >0$ on $M_0$ and $Q_1>0$ at $\left( x_0,t_0\right)$. This completes the proof. □

#### Corollary 5.1

We have

5.12 $$\begin{aligned} \left\| \varphi \right\| _{C^2 \left( \mathbb {S}^n \times [0, T)\right) } \le C, \end{aligned}$$

where *C* is a positive constant depending only on $M_0$.

#### Proof

Recall Formula (2.12),

$$\begin{aligned} D^2 \varphi = A[\varphi ] + \left( \psi -\varphi \right) \sigma . \end{aligned}$$

In view of Formula (2.15), the $C^0$ estimate (5.1) and the lower bound of the shifted principal curvatures in (5.8) imply that $A[\varphi ] < C \sigma$ along the Eq. (3.1). On the other hand, we know from the $C^0$ and $C^1$ estimates that $\left| \psi - \varphi \right|$ is uniformly bounded. Hence, the $C^2$-norm of φ is uniformly bounded on $\mathbb {S}^n \times [0, T)$. □

We will show in the following that, along the flow (1.2), the shifted principal curvatures of $M_t$ will not blow up in any finite time interval. The proof is based on an estimate of the shifted principal curvatures in the proof of [14, Proposition 7.4].

#### Lemma 5.6

Assume that T<∞. Along the flow (1.2), we have

5.13 $$\begin{aligned} \tilde{\kappa }_i \le C_T, \quad i=1, \ldots , n, \end{aligned}$$

where $C_T$ is a positive constant depending only on $M_0$ and *T*.

#### Proof

Denote by $\zeta \left( x,t\right)$ the largest shifted principal curvature at $\left( x, t\right) \in M \times [0, T)$. It was proved in [14, Eq. (7.13)] that, at the spatial maximum point of $\zeta \left( \cdot , t\right)$,

5.14 $$\begin{aligned} \frac{\partial }{\partial t} \zeta&\le - \frac{\lambda '-\tilde{u}}{F^2} \left( F+1\right) \zeta ^2 + \left( \lambda '+\left( \lambda '-\tilde{u}\right) \left( (n-m+1)-F^{-1} \right) \right) \nonumber \\&\zeta + \left( n-m+2\right) \left( \lambda '-\tilde{u}\right) \nonumber \\&\le \left( \lambda '+ (n-m+1) \left( \lambda '-\tilde{u}\right) \right) \zeta + \left( n-m+2\right) \left( \lambda '-\tilde{u}\right) . \end{aligned}$$

The $C^0$ estimate in (5.6) yields uniform upper bounds for the coefficient of ζ and the zero-order term in (5.14). Applying the maximum principle, we have that ζ is bounded from above by a constant $C_T$ depending only on $M_0$ and *T*. □

It follows from (5.13) and the Formula (2.15) that the strict positivity of A[φ] is preserved along the Eq. (3.1).

#### Corollary 5.2

We have

5.15 $$\begin{aligned} A [\varphi ]>C_T^{-1}>0 \quad \text {on}~ \mathbb {S}^n \times [0, T), \end{aligned}$$

where $C_T$ is a positive constant depending only on $M_0$ and *T*.

Consequently, the function $\log \varphi (\cdot ,t)$ remains the horospherical support function of a strictly h-convex hypersurface $M_t$. This preserves the equivalence between the scalar equation (3.1) and the geometric flow (1.2).

## Proof of the Results

### Proof of Theorem 1.1

First, we prove the long-time existence of the flow (1.2). The short-time existence of the solution to (3.1) follows from the positivity of the tensor $A[\varphi (\cdot , 0)]$ on $\mathbb {S}^n$. It follows from (5.12) that the $C^2$-norm of φ is uniformly bounded from above. Moreover, this together with (5.15) implies that the eigenvalues of A[φ] are in a compact subset of $\Gamma ^+$ for all $\left( z, t\right) \in \mathbb {S}^n \times [0,T)$ when T<∞. Thus, Eq. (3.1) is uniformly parabolic on $\mathbb {S}^n \times [0,T)$ for any finite *T*. Applying the Krylov theory for the $C^{2, \alpha }$ estimate of φ and the standard parabolic Schauder estimate for higher order derivatives, we obtain the long-time existence of the smooth, strictly h-convex solution to the Eq. (3.1). This means that the flow (1.2) exists for a long time.

Next, we prove the convergence of the flow (1.2). Let $B \left( Y_0, \delta \right)$ be a geodesic ball lying inside $\Omega _0$. By the standard comparison principle and Proposition 4.1, the evolving geodesic ball $B \left( Y\left( t\right) , \delta \right)$ lies inside $\Omega _t$ for all t>0. Then, there exists a $T_0 >0$ depending only on $Y_0$ and δ so that $M_{T_0}$ is a star-shaped and strictly h-convex hypersurface with the ball $B\left( \mathcal {O}, \delta /2\right)$ lying inside $\Omega _{T_0}$. We can then view $M_{T_0}$ as the initial hypersurface and adapt the result proved in [14, Sect. 8.3] to obtain that $M_t$ converges exponentially fast to a geodesic sphere centered at the origin as $t \rightarrow \infty$. This completes the proof.

### Proof of Theorem 1.2 and Corollary 1.1

Let $\mathcal {V} = \int _{M} \left( X-\nu \right) p_m \left( \tilde{\kappa }\right) \, d\mu$. Since $\langle X-\nu , X-\nu \rangle _1= 0$ and $\lambda '-\tilde{u}\ge \lambda '-\lambda >0$ on *M*, we have that for any X∈M, the vector X-ν lies on the future light cone $\partial \mathcal {C}$, where

6.1 $$\begin{aligned} \mathcal {C}:= \left\{ X \in \mathbb {R}^{n+1,1};~ x_{n+2}> \left| x\right| \right\} . \end{aligned}$$

Moreover, since the horospherical Gauss map $G: M \rightarrow \mathbb {S}^n$ is a diffeomorphism, the vectors $\left\{ X-\nu ;~ X \in M\right\}$ span $\partial \mathcal {C}$. Then the strict h-convexity of *M* implies that $\mathcal {V}$ is future timelike, and thus $\tilde{\mathcal {V}}:= \mathcal {V}/ N \left( \mathcal {V}\right)$ is on $\mathbb {H}^{n+1}$. Modulo a suitable Lorentzian transformation, we can assume without loss of generality that $\tilde{\mathcal {V}} = E_{n+2}$, and then

$$\begin{aligned} N \left( \mathcal {V}\right) = \int _M \left( \lambda '-\tilde{u}\right) p_{m} \left( \tilde{\kappa }\right) \, d\mu . \end{aligned}$$

Set $M_0= M$ as the initial hypersurface of the flow (1.2). Note that we do not need to verify whether $\tilde{\mathcal {V}}$ (i.e., the origin) lies inside $\Omega _0$. Recalling the following monotone quantities along the flow (1.2) proved in [14, Sect. 9.1]:

$$\begin{aligned} \frac{d}{dt} \widetilde{W}_m \left( \Omega _t\right) = 0, \quad \frac{d}{dt} \int _{M_t} \left( \lambda '-\tilde{u}\right) p_m \left( \tilde{\kappa }\right) \, d\mu _t \le 0, \end{aligned}$$

and equality holds in the second inequality if and only if $M_t$ is a geodesic sphere centered at $\tilde{\mathcal {V}}$. By the long-time existence and the convergence results of the flow (1.2) in Theorem 1.1, we have

$$\begin{aligned} N \left( \mathcal {V}\right) \ge \tilde{h}_m \circ \tilde{f}_{m}^{-1} \left( \widetilde{W}_m \left( \Omega \right) \right) , \end{aligned}$$

with equality if and only if Ω is a geodesic ball centered at $\tilde{\mathcal {V}}$. This completes the proof of Theorem 1.2.

To prove the Corollary 1.1, we first show that $\tilde{\mathcal {V}} = Y$ if Ω is a domain symmetric with respect to *Y*. For this identity, we can assume without loss of generality that $Y = E_{n+2}$. Consider the projection $\pi : \mathbb {R}^{n+1,1} \rightarrow \mathbb {R}^{n+1}$ defined by $\pi \left( X\right) = x$. We obtain from the symmetry of Ω that $\int _M \pi \left( X-\nu \right) p_m \left( \tilde{\kappa }\right) \, d\mu =0$, which means $\mathcal {V}$ is parallel to *Y*, and thus $\tilde{\mathcal {V}} =Y$.

Using the identity $\tilde{\mathcal {V}} =Y$, (2.5) and (2.3), we have

$$\begin{aligned} \int _M \left( \lambda '-\tilde{u}\right) p_m \left( \tilde{\kappa }\right) \, d\mu&= - \langle \mathcal {V}, E_{n+2} \rangle _1 = \cosh d_{\mathbb {H}^{n+1}} \left( \mathcal {O}, Y\right) N \left( \mathcal {V}\right) . \end{aligned}$$

Combining the above formula with the inequality (1.5), we obtain (1.6). This completes the proof of Corollary 1.1.

### Proof of Proposition 1.1

Let $\mathcal {W} = \int _M X \mathscr {F} \, d\mu$. Since the cone $\mathcal {C}$ defined in (6.1) is convex, we see that $\mathcal {W}$ is future timelike. Set $\tilde{\mathcal {W}}= \mathcal {W}/ N \left( \mathcal {W}\right)$. Using (2.3), we have

6.2 $$\begin{aligned}&\int _M \lambda ' \mathscr {F} \, d\mu = -\langle \mathcal {W}, E_{n+2} \rangle _1 = N \left( \mathcal {W}\right) \cosh d_{\mathbb {H}^{n+1}} \left( \mathcal {O}, \tilde{\mathcal {W}}\right) , \end{aligned}$$

6.3 $$\begin{aligned}&N \left( \mathcal {W}\right) = - \langle \mathcal {W}, \tilde{\mathcal {W}} \rangle _1 = \int _{M} \cosh d_{\mathbb {H}^{n+1}} \left( X, \tilde{\mathcal {W}}\right) \mathscr {F} \, d\mu . \end{aligned}$$

Consider the cone generated by Ω in $\mathbb {R}^{n+1,1}$:

$$\begin{aligned} \mathscr {C} = \left\{ tX \in \mathbb {R}^{n+1,1};~ X \in \Omega , \, t \in (0, +\infty )\right\} . \end{aligned}$$

It is a common fact that the convexity of Ω is equivalent to the convexity of $\mathscr {C}$, as the geodesic connecting two points $X, Y \in \mathbb {H}^{n+1}$ is given by the intersection of the 2-dimensional cone spanned by *X* and *Y* in $\mathbb {R}^{n+1,1}$ with the hyperboloid model, see e.g. [24, Sect. 3.2]. Since $\mathscr {F}$ is positive and $\partial \Omega$ spans $\partial \mathscr {C}$, we have $\mathcal {W} \in \mathscr {C}$ and thus $\tilde{\mathcal {W}} \in \Omega$. Let $X_0 = \tilde{W} \in \Omega$. Combining (6.2) with (6.3), we obtain (1.7). This completes the proof.

## Data Availability

Not applicable.
